# Place du repérage isotopique peropératoire dans la prise en charge de l'ostéome ostéoïde

**DOI:** 10.11604/pamj.2014.19.158.4919

**Published:** 2014-10-16

**Authors:** Monsef Boufettal, Amina Haddam, Issam Lalya, Rachid El Zanati, Mustapha Mahfoud, Ahmed El Bardouni, Mohamed Saleh Berrada, Nouzha Benraïs, Moradh El Yaacoubi

**Affiliations:** 1 Service de Traumatologie et Orthopédie, Centre Hospitalier Universitaire Avicenne, Université Mohammed V, Rabat, Maroc; 2 Service de Médecine Nucléaire, Centre Hospitalier Universitaire Avicenne, Université Mohammed V, Rabat, Maroc; 3 Service de Radiothérapie, Hôpital Militaire d'Instruction Mohammed V, Rabat, Maroc

**Keywords:** Chirurgie, marges saines, ostéome ostéoïde, repérage isotopique, surgery, healthy margins, osteoid osteoma, isotope marking

## Abstract

L'ostéome ostéoïde est une tumeur osseuse bénigne. Le diagnostic est clinique et radiologique, et le traitement consiste en l'exérèse chirurgicale totale avec des marges saines. Nous rapportons 05 cas d'ostéome ostéoïde opérés avec succès à l'aide d'un repérage isotopique peropératoire. Nous précisons les avantages de cette technique dans l'orientation de l'exérèse chirurgicale ainsi que la confirmation de son caractère radical.

## Introduction

L′ostéome ostéoïde est une tumeur bénigne ostéoblastique relativement rare, constituée d′une petite lésion de tissu charnu et vascularisé, appelé nidus, cernée d′une ostéocondensation réactionnelle. La tumeur est petite, à potentiel évolutif limité mais à douleur disproportionnelle [1]. Le traitement est exclusivement chirurgical, et la résection complète de la tumeur est le seul garant de l′absence de récidive [2]. La difficulté du geste opératoire émane de la localisation de la tumeur, de ses rapports anatomiques mais surtout desa petite taille; ce qui justifie l′intérêt de la technique du repérage isotopique peropératoire. Cette étude vient en continuité desdeux cas précédemment publiés par notre équipe [3, 4], et se propose de rapporter notre expérience, tout en décrivant la technique de repérage isotopique peropératoire de l′ostéome ostéoïde à propos de cinq cas, ses avantages, les perspectives d′avenir ainsi que sa place parmi les autres moyens de repérage peropératoire.

## Méthodes

### Eligibilité

Après l′approbation du comité d′éthique de la faculté de médecine et de pharmacie, les dossiers des patients ayant bénéficié d'un traitement chirurgical d'O.O avec repérage isotopique peropératoire, au service de chirurgie orthopédique, ont été revus rétrospectivement. Les patients ayant un recul inférieur à 12 mois, ont été exclus de l’étude. Ainsi notre étude s'est étalée de 2008 à 2010.

### Caractéristiques des patients

La technique de repérage isotopique peropératoire de l′ostéome ostéoïde a été programmée pour 5 patients colligés au service de chirurgie orthopédique, ils étaient tous de sexe masculin, avec un âge moyen de 19,6 ans, sans antécédents pathologiques particuliers, le délai moyen de consultation était de 16,5 mois, et la symptomatologie clinique était dominée par des douleurs type inflammatoire d′apparition nocturne. La localisation de cette lésion était préférentielle au niveau des membres inférieurs (Tableau 1). Tous nos patients ont bénéficié d′examens complémentaires notamment des radiographies standards, une scintigraphie osseuse, des compléments morphologiques centrés, en l′occurrence la tomodensitométrie.

**Tableau 1:** Récapitulatif des patients admis au service de médecine nucléaire et suivis pour l’étude de l'apport du repérage isotopique peropératoire pour l'optimisation du traitement de l'ostéome ostéoïde

	Age	Localisation	Clinique
Patient 1	21 ans	Tiers supérieur du tibia gauche	Douleur
Patient 2	13 ans	Tiers moyen du fémur gauche	Douleur
Patient 3	25 ans	A cheval des tiers supérieur et moyen du fémur droit	Douleur
Patient 4	22 ans	Tiers moyen du fémur droit	Douleur + tuméfaction
Patient 5	17 ans	Le col fémoral droit	Douleur

### La molécule de marquage

La molécule utilisée est l'HydroxyMéthyl Di Phosphonate (HMDP) qui est un biphosphonate traceur se fixant sur la trame osseuse. L'HMDP est radiomarqué au technétium 99m (HMDP-99Tc) émetteur des rayonnements gamma à 140 keV.

### La sonde de détection utilisée est de type Gammasonic

C'est un détecteur à scintillation, courbé, polyvalent de 14 mm de diamètre conçu pour la détection des isotopes dont l’énergie des photons se situe entre 40 et 511 keV, muni d'un collimateur relié au boîtier électronique par un câble flexible de 3,5 m. Les résultats sont donnés en coups par seconde (cps) par affichage numérique. Cette sonde fonctionne à température ambiante et à celle du corps humain, elle est insensible aux chocs et aux perturbations électromagnétiques (bistouri électrique). Elle présente l'avantage d’être au contact du foyer fixant et d’être dirigée suivant l'incidence qui fournit le taux de comptage le plus élevé, celui- ci sera affiché et sonore. On a considéré comme signal, le foyer de l'OO et, comme bruit de fond, l'activité de l'os sain loin du point (ou des points) hyperfixant(s).

### Description de la technique

**Figure 1:**
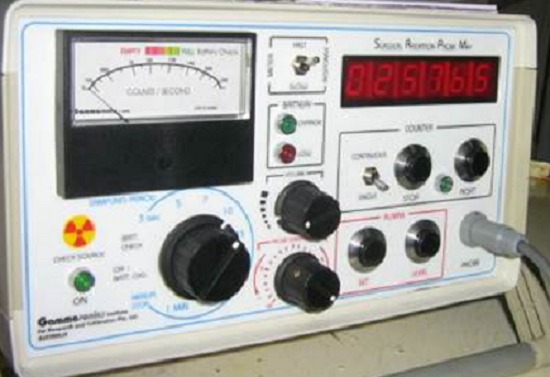
Boitier électronique d'enregistrement des comptages

**Figure 2:**
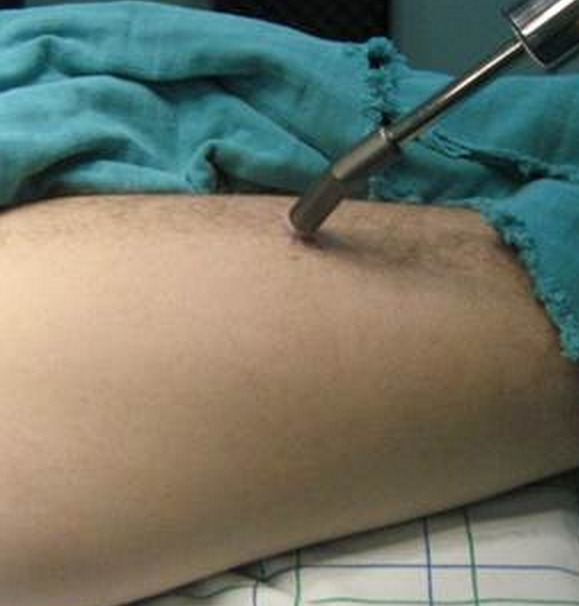
Le repérage isotopique percutané

**Figure 3:**
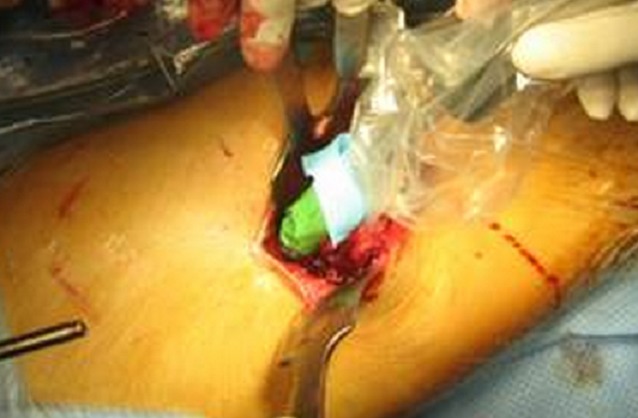
La sonde de détection isotopique Gamma sup au contact de l'os pour repérer le nidus

**Figure 4:**
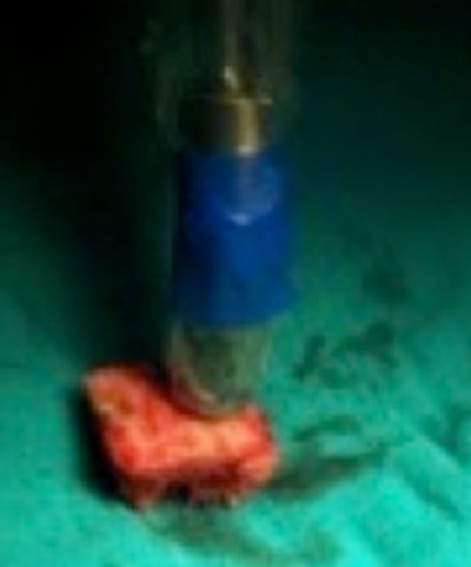
Comptage des cps du fragment osseux réséqué

La technique consistait à administrer pour chacun de nos patients trois, ou quatre et jusqu′à sept heures avant l′intervention 8 MBq/Kg d′HMDP-99mTc par voie intraveineuse. Pour s′assurer de la fixation du traceur sur la lésion à traiter, un premier repérage scintigraphique est réalisé avec enregistrement corps entier par la gamma caméra. Au bloc opératoire, la radiodétection s′effectue en étroite collaboration entre chirurgien et médecin nucléaire avec un aide qui note les comptages sur le boîtier électronique (Figure 1). Un repérage percutané pour le centrage de la voie d′abord sur la lésion (Figure 2) est suivi, après incision et abord de l′os, d′un repérage du point de fixation le plus important et des enregistrements sont alors effectués de centimètre en centimètre dans les quatre points cardinaux (Figure 3). Ceci permet d′établir une cartographie du radio-marquage autour du point de fixation maximal, avant et après résection de la lésion. Le comptage du nombre de coups par seconde enregistré sur le fragment osseux enlevé sera également effectué (Figure 4). En somme, la sonde de repérage isotopique est employée dans les différents temps de l′intervention chirurgicale. Elle permet d′abord de localiser l′ostéome ostéoïde, puis de guider l′étendue de la résection chirurgicale et surtout elle vérifie la qualité de celle-ci par la disparition totale en fin d′intervention de tout foyer anormal d′hyperfixation. La marche a été autorisée sans appui pour une durée de 30 à 45 jours.

## Résultats

Tous les patients ont vu disparaitre leur douleur le lendemain de l′intervention. Les résultats numériques des enregistrements sont rapportés dans le (Tableau 2). Dans tous les cas, la sonde a permis d′identifier la lésion comme le point de la plus forte fixation du radiotraceur parfaitement reproductible géographiquement à chaque nouveau comptage et a même permis dans le cas du patient 1, de rectifier la localisation du nidus qui n′était pas tout à fait au summum d′une boursouflure de la corticale. A partir du point d′hyperfixation, le déplacement d′un centimètre de la sonde a toujours fait chuter le nombre de cps, (en moyenne une diminution de 26,2%). La résection du nidus a toujours fait chuter le nombre de cps, en moyenne de 46,2% (34 à 55%).

**Tableau 2:** Les résultats numériques des nombres de cps enregistrés lors de l'intervention

	Patient 1	Patient 2	Patient 3	Patient 4	Patient 5
Le point osseuxle plus hyperfixant	2 3106	4398	32917	34764	38279
La moyenne à 1 cm du point hyperfixant dans les quatre points cardinaux	17815	2736	29802	30240	20319
Sur le lit de résection	15200	1995	20730	16300	18273

Aucun patient ayant bénéficié du repérage isotopique peropératoire pour la résection de l′ostéome ostéoïde n′a eu besoin d′un matériel d'ostéosynthèse. L′examen anatomopathologique a confirmé dans tous les cas l′ostéome ostéoïde avec présence du nidus sur la pièce de résection. Aucune récidive n′a été note chez nos patients après recul moyen de 40 mois.

## Discussion

L'ostéome ostéoïde est la plus fréquente des tumeurs bénignes à histogenèse osseuse, cette tumeur de petite dimension (< 1 cm) se caractérise par une structure spécifique: le nidus, constitué d'un tissu ostéoïde et entouré par une ostéocondensation réactionnelle. Elle représente 2 à 3% des tumeurs osseuses et 10 à 20% des tumeurs bénignes. Elle se caractérise par une prédominance masculine (deux à trois pour un) et par sa survenue entre la seconde enfance et l’âge adulte. Le diagnostic se fait le plus souvent par des radiographies simples ou par la tomodensitométrie. Parfois, cette tumeur n'est visible qu’à la scintigraphie osseuse sous forme d'une zone d'hyperfixation («hot spot») [5], la sensibilité est proche de 100%, et la littérature ne mentionne pas de faux négatifs [6].

La littérature rapporte de nombreuses possibilités de traitement de l'ostéome ostéoïde [7, 8], mais aucune thérapeutique, autre que la chirurgie ne semble avoir d′action sur L′ostéome ostéoïde. La résection du seul nidus est nécessaire et suffisante à la guérison, mais sa simple destruction mécanique ou physicochimique est aussi possible [9, 10]. La prise des AINS au long cours a été proposée dans l'attente de la disparition «spontanée » de l'ostéome ostéoïde, ce qui marque l'arrêt de ce traitement médical uniquement symptomatique [11], mais la plupart des patients, ne tolérant pas la douleur, abusent des AINS qui sont, quant à eux, pourvoyeurs d'effets secondaires notamment gastro-intestinaux [12]. La problématique du traitement chirurgical de l'ostéome ostéoïde vient de ses caractéristiques histologiques: petite taille de la seule zone pathologique, situation anatomique, difficulté de localisation en cours d'intervention, alors que son succès du traitement dépend étroitement d'une bonne localisation et de l'ablation ou la destruction complète du nidus. En effet, il n'est pas nécessaire d'enlever toute la réaction osseuse sclérotique pour cerner la pathologie [13]. Une exérèse trop limitée risque de laisser en place une partie du nidus et d'entraîner une récidive. Au contraire, une exérèse trop large visant à coup sûr le nidus risque d'entraîner une fracture ou des troubles de croissance, comme cela a bien été souligné par Boulaya [14].

Pour optimiser le traitement chirurgical, plusieurs techniques ont été élaborées. Elles permettent soit un meilleur repérage de la lésion soit un meilleur contrôle de son exérèse, soit les deux [15]. Parmi ces techniques, la méthode de repérage isotopique peropératoire est réputée pour sa grande sensibilité [2, 15–23], bénéfique pour le patient, lui épargnant une plus grande résection par les méthodes « classiques », facilitant ainsi une reprise rapide de l'activité. Rinsky et al. [16] rapportent, pour la première fois en 1980, l'intérêt du repérage isotopique peropératoire pour la résection d'un OO d'une vertèbre. Depuis, les données de la littérature sur l'usage peropératoire de la scintigraphie osseuse font état de courtes séries [2, 17–21] ou de cas sporadiques [15, 22–25]; les analyses quantitatives restent assez rares.

Le repérage isotopique peropératoire présente plusieurs avantages par rapport aux autres méthodes de repérage, notamment scannoguidé: soulignons d'abord l'innocuité de la méthode pour le malade et pour le personnel [26]; c'est une méthode simple: l'appareillage est peu encombrant, la sonde facilement manipulable tout en respectant les règles de la chirurgie orthopédique, les contraintes de balayage, sonde perpendiculaire à la surface explorée, déplacement lent sont faciles à respecter; méthode fiable: Le repérage a été efficace dans tous nos cas, comme en témoigne l'absence de récidive après un recul suffisant; méthode précise de localisation, de l'ordre de quelques millimètres, permettant des exérèses très mesurées; méthode garantissant l'efficacité du geste: ablation totale du nidus vérifiée par la disparition du foyer d'hyperfixation; méthode n'allongeant pas de manière significative le temps d'intervention [27]; dans les localisations dont l'abord est difficile ou dont l'ablation fragilise le segment osseux concerné comme au niveau du col du fémur ou au niveau du rachis, la détection de la radioactivité apporte une précision dans le geste et une sécurité dans l′excision [26]

Le repérage isotopique peropératoire de l′ostéome ostéoïde est moins irradiant que le repérage scannoguidé; cette technique reste insuffisante car elle ne permet pas toujours de distinguer le nidus de la réaction osseuse qui l′entoure. Dans le cas où le nidus est profond et qu′aucune réaction osseuse n′apparait sur la surface, le repérage radiologique peropératoire devient totalement inefficace. Principales difficultés du repérage scintigraphique per-opératoire [28]: Artéfacts causés par le bistouri électrique; orientation de la sonde par rapport à la surface à analyser: la sonde doit être perpendiculaire au plan à analyser. En cas d'impossibilité, la sonde peut être placée dans le plan le plus adéquat. Dans ce cas, toutes les mesures se feront dans ce plan et le nucléariste en tiendra compte pour ses calculs; déplacement lent de la sonde afin d’évaluer au mieux la croissance ou la décroissance du signal; la variabilité de l’épaisseur et de la nature des différentescouches de tissus, scléreux et calcifié, qui séparent la lésion de la sonde; la présence de concentration élevée de produit de contraste dans les organes adjacents ou sous-jacents, telle la vessie; Présence de sang dans le champ opératoire; une taille de l'os à opérer inférieure ou égale à la taille dela sonde.

Ainsi la technique nécessite une étroite collaboration entre isotopiste et le chirurgien, et doit être rigoureuse. La sonde doit être maintenue perpendiculairement à la surface osseuse explorée et, pour les lésions autour du bassin, la vessie doit être vide, voire sondée, pour éviter tout bruit de fond parasite. La proximité d'un cartilage de croissance fertile chez l'enfant, rend prudente l'interprétation vu la fixation intense à ce niveau [4].

Concernant les caractéristiques de la sonde utilisée, la plupart des travaux rapportés font état de sondes avec un scintillateur d′iodure de sodium activé au Thallium, notre sonde présente un capteur d′iodure de Césium dopé au Thallium qui présente l′avantage de détecter les lésions de faible fixation du traceur et surtout un temps très faible (quelques secondes) pour chacune des mesures. Cependant, quelque soit le capteur, il est indispensable pour la précision du repérage, d′utiliser la sonde collimatée malgré l′inconvénient d′augmenter le diamètre de la sonde.

## Conclusion

Le repérage isotopique peropératoire pour la résection de l'ostéome ostéoïde est un outil particulièrement fiable. En respectant les impératifs techniques, il permet la localisation précise à moins d'un centimètre des lésions de petite taille hyperfixantes et autorise ainsi à une résection économe, tout en garantissant un excellent contrôle local.
